# Correlation Between Fungal and Bacterial Populations in Periodontitis Through Targeted Sequencing: A Pilot Study

**DOI:** 10.3390/jcm14186418

**Published:** 2025-09-11

**Authors:** Jacob Ayers, Jennifer Chinnici, Amarpreet Sabharwal, Pinar Emecen-Huja, Hua-Hong Chien, Shilpi Joshi, Abhiram Maddi

**Affiliations:** 1 Division of Regenerative Sciences and Periodontics, Department of Advanced Specialty Sciences, James B Edwards College of Dental Medicine, Medical University of South Carolina, Charleston, SC 29425, USA; jlayers@g.clemson.edu (J.A.); emecenh@musc.edu (P.E.-H.); chienh@musc.edu (H.-H.C.); 2 Roswell Park Cancer Center, Buffalo, NY 14203, USA; chinnici@roswellpark.org; 3 Division of Periodontics, Schulich School of Medicine and Dentistry, Western University, London, ON N6A 5C1, Canada; amarpreet.sabharwal@schulich.uwo.ca; 4 Department of Periodontics, School of Dental Medicine, University at Buffalo, Buffalo, NY 14215, USA; joshis@neighborhealth.com

**Keywords:** periodontitis, microbiome, mycobiome, fungi, *Candida albicans*, red complex bacteria, saliva

## Abstract

**Background and Objective:** The oral microbiome plays an important role in oral health and disease, including periodontitis, which affects about 40% of the adult population in the United States. Bacterial pathogens have been well studied and documented in their relationship with periodontitis; however, the role of fungi in periodontitis is still unclear. The purpose of this study is to determine the relationship of specific fungal species with periodontal pathogenic bacteria in healthy, mild periodontitis, and severe periodontitis patients. **Methods:** In this study, human participants were recruited, and saliva samples were collected. Twelve participants representing periodontal health (*n* = 2), mild periodontitis (*n* = 3), and severe periodontitis (*n* = 7) were included. Salivary samples were sequenced for analysis of their mycobiome (ITS sequencing) and microbiome (16s RNA sequencing). **Results:** A total of 375 species of bacteria and 39 species of fungi were identified among all samples. Clustering was observed for bacteria in healthy and mild periodontitis, but more variability was observed in the severe periodontal disease group. Variability was observed for fungi among all samples and groups. Red complex bacteria were negatively correlated with Candida species for the disease groups, although the correlation was not statistically significant. A significant correlation was observed between red-complex bacteria in the severe periodontal disease group. Additionally, a significant correlation was observed among Candida species in all groups. **Conclusions:** This pilot study simultaneously processed saliva samples for microbiome and mycobiome sequencing and found a trend towards negative correlation between Candida species and red complex bacteria.

## 1. Introduction

Periodontal disease is a major oral disease of microbial etiopathogenesis that affects 40% of adults who are 30 years or older in the United States [1]. Additionally, the prevalence of periodontal disease increases, with age with more than 60% for adults over 65 years of age being affected [1]. Periodontitis leads to the destruction of the supporting tissues, including periodontal ligament, alveolar bone, and connective tissue, around teeth and is a major cause of tooth loss [2]. Additionally, periodontal disease can worsen systemic conditions, including diabetes [3,4]. Several periodontal pathogens have been associated with systemic conditions like cardiovascular disease, strokes, cancer, Alzheimer’s disease, and rheumatoid arthritis, increasing the significant impact of periodontal disease on systemic disease [5]. More recently, fungal mycobiota was isolated in a pan-cancer analysis, and *C. albicans* was positively associated with head and neck cancer and gut cancer with reduced survival rates [6].

Periodontal disease is a chronic multifactorial inflammatory disease associated with dysbiotic biofilms and characterized by the progressive destruction of the periodontium [7]. While the oral microbiome is very diverse, previous studies have shown a strong association between certain oral bacteria and periodontal disease. When separated into clusters, the red complex bacteria (*Porphyromonas gingivalis*, *Tannerella forsythia*, and *Treponema denticola*) are associated with periodontal disease severity as measured by pocket depth and bleeding on probing [8]. These red complex bacteria have been found to be significantly more prevalent in current smokers and have been found in higher proportions of peri-implantitis samples [9]. Elevated levels of *P. gingivalis* have been found around mobile teeth [10], and its presence in dental biofilm following periodontal treatment can be indicative of progressive bone loss [11]. *T. forsythia* and *P. gingivalis* have been found to be strong bacterial markers for periodontal disease and are infrequently cultured from patients without periodontal bone loss [12]. The elimination of red complex bacteria results in improved periodontal outcomes, further strengthening their association with periodontal disease.

An early study on the oral mycobiome of healthy participants using panfungal internal transcribed spacer (ITS) primers had shown that *Candida* sp. was most frequently isolated, but the oral mycobiome was surprisingly diverse for fungal species [13]. Another study that compared oral mycobiomes between periodontally healthy and periodontitis participants confirmed the diverse oral mycobiome and a trend towards higher median relative abundance in participants with periodontal disease [14]. A recent systematic review on the association of *Candida* sp. with periodontal disease concluded a strong association between presence of *Candida* sp. while recognizing that pathogenic mechanisms of *Candida* sp. in periodontal disease are not established [15]. *Candida albicans* is associated with five times higher odds [16] of early childhood dental caries. Further, the presence of *C. albicans* has been shown to be associated with a highly acidogenic and acid-tolerant bacterial community and an increased abundance of *Streptococcus mutans* in dental biofilm [17]. Physical, metabolic, and chemical interactions between fungi and bacteria have been proposed to play a role in health and disease. Further, there is evidence that fungi influence bacterial diversity and potentially contribute to the pathogenesis of oral diseases [18].

The availability of microbial ecology markers for bacteria (16S) and fungi (ITS) can aid in simultaneous analysis of oral microbiome and mycobiome for the accurate study of correlations and variations in samples. Dissimilarities between bacterial and fungal cell structure and methods used to disrupt these cells for genomic DNA extraction and sequencing can make a simultaneous analysis of oral microbiome and mycobiome challenging [19]. In this study, we developed methods for this simultaneous analysis and conducted a pilot survey of salivary microbiome and mycobiome to explore bacterial and fungal ecology in periodontal health and disease.

## 2. Materials and Methods

Saliva samples were obtained from the University at Buffalo (UB) Microbiome Center, Buffalo, NY. The samples were selected at random from a larger epidemiologic study (DM-RCT study, STUDY00000962) that included healthy subjects, subjects with periodontal disease only, and subjects with periodontal disease and type II diabetes approved by the Institutional Review Board of the University at Buffalo, Buffalo, NY. For our study, healthy subjects and subjects with periodontal disease were included. The subjects of our study did not have any underlying systemic condition. Subjects of the DM-RCT study were subjected to periodontal examination and classified with diagnosis of periodontitis as mild or severe, as described previously [20]. A total of 12 samples were selected from participants with periodontal health (*n* = 2), mild periodontitis (*n* = 3), and severe periodontitis (*n* = 7).

***Saliva collection:*** Participants abstained from brushing teeth, chewing gum, and eating or drinking for at least 1.5 h before saliva collection. Each participant rinsed well with tap water for 30 s, expectorated the water, and waited for 2 min before saliva collection. Saliva accumulated in the floor of the mouth for the 60s was emptied into collection tubes and repeated 14 times in a 15 min collection period. An average of 5 mL of unstimulated saliva was collected per participant. From the 5 mL of unstimulated saliva, 250 µL was allocated per tube for further analysis. The samples were labeled and stored in −80 °C until used.

**Figure 1:**
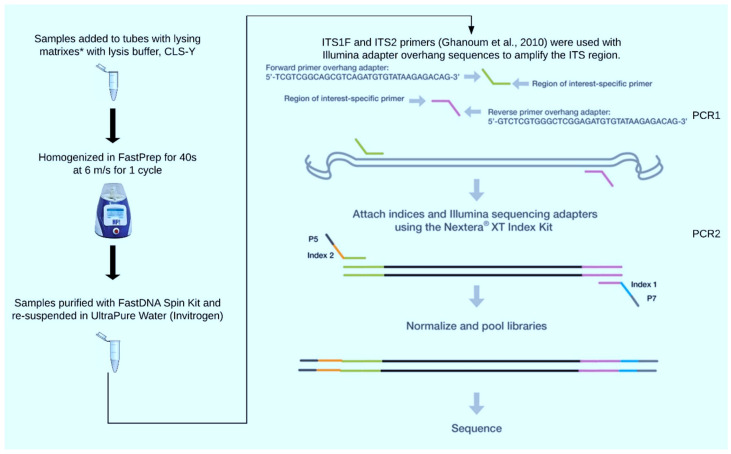
Overview of mycobiome sequencing protocol [13].

***Selection of lysing matrix*:** For selection of a lysing matrix, saliva samples were collected from twenty-five healthy volunteers using the same method as described above. However, these saliva samples were pooled and subjected to the genomic DNA extraction method as described below (and summarized in Figure 1). The three lysing matrix choices included A (MP Biomedicals, Santa Ana, CA, USA), F (MP Biomedicals, Santa Ana, CA, USA), and L. Lysing matrix L was composed of glass beads measuring 425–600 µm in diameter (Sigma-Aldrich, St. Louis, MO, USA) in 2 mL bead tubes with caps (VWR International, Radnor, PA, USA). Lysing matrix A was chosen for use with saliva samples from individual study participants.

***Genomic DNA extraction*:** Genomic DNA extraction was performed as previously described [19]. Briefly, saliva samples were centrifuged at 5000× *g* for 10 min at 4 °C to obtain pellets. The supernatant was removed, and the pellets were resuspended in 200 µL of AL lysis buffer (Qiagen, Hilden, Germany). The resuspended pellets were transferred to FastPrep tubes, and an equal volume of sterile glass beads (425–500 µm) was added (Sigma-Aldrich, St. Louis, MO, USA). Three rounds of homogenization were performed for 30s each at 6 m/s in a FastPrep machine with samples placed on ice for 5 min intervals between each round. Thereafter, samples were transferred to matrix tubes with lysing matrix A and were agitated for 1 cycle of 40 s at 6 m/s and 200 µL of centrifuged lysate was transferred to a sterile tube. Genomic DNA extraction was performed from this lysate as per manufacturer’s instructions (FastDNA SPIN Kit, MP Biomedicals, Santa Ana, CA, USA), eluted into 100 µL of UltraPure DNAse/RNAse free water (Thermo Fisher Scientific, Grand Island, NY, USA), and stored at −20 °C.

***Genomic DNA quantity assessment*:** DNA yield was determined with Quant-iT PicoGreen ds DNA assay kit (Thermo Fisher Scientific, Grand Island, NY, USA) using the F200 fluorescence microplate reader as described previously [21]. The supplied lambda DNA standard was diluted with 1x TE, pH 8 (VWR International, Radnor, PA, USA) buffer to create a serial dilution series of seven dilutions of known DNA concentrations ranging from 0.002 ng/mL to 2 ng/mL to construct a standard curve. The standards were added to a 96 well, black, flat bottom microplate (Tecan, Zurich, Switzerland) in duplicate. Two wells of 1x TE, pH 8 buffer were used as blanks. Further, 49 µL of 1x TE buffer and 1 µL of each sample of DNA were added and mixed well, followed by addition of fluorescent dye to all wells and incubated at room temperature for 5 min. The quantitation assay was read on a Tecan F200 fluorescence microplate reader using an excitation/emission setting of 485/20 and 528/20.

***PCR amplification*:** PCR amplification of genomic DNA was performed as previously described [13,19]. For comparison of lysing matrices, genomic DNA was subjected to PCR amplification of the fungal internal transcribed spacer 1 (ITS 1) region. For individual saliva samples, PCR amplification was performed for both 16S rRNA and ITS 1. For each sample, genomic DNA extraction was performed in duplicates, and following PCR1, these samples were pooled in PCR2.

PCR1: ITS1F and ITS1R primers were used to amplify ITS1 region and primers for V1-V3 and V3-V4 hypervariable regions were used for amplification of the 16S rRNA. Each 25 µL reaction contained 10.5 µL (about 12.5 ng/reaction) of DNA template, 0.2 µM ITS1F primer, 0.2 µM ITS2 primer, and 12.5 µL 2x Kappa HiFi HotStart Ready Mix (Kappa Biosystems, Wilmington, MA, USA). UltraClean PCR Water (Qiagen, Hilden, Germany) and CLS-Y Buffer were used as negative controls. ZymoBIOMICS Microbial Community Standard (Zymo Research, Irvine, CA, USA) was used as positive control, and PCR water was used as negative control. The primer sequences and thermocycling conditions for PCR are provided in Table 1.

PCR1 clean up: PCR 1 reactions were purified using AMPure XP beads (Beckman Coulter, Pasadena, CA, USA). After being vortexed, 80 µL of beads were added to each well on a 96 well plate (Thermo Scientific, Grand Island, NY, USA). The PCR reactions were added to the beads and mixed a few times using a pipette. The samples were allowed to incubate for 5 min at room temperature and then placed on a magnetic stand until the supernatant appeared clear. The supernatant was removed, and the beads were washed twice with 200 µL of 80% ethanol. The ethanol was removed, and the beads were allowed to dry for 20 min. The plate was removed from the magnetic stand, and the beads were re-suspended in 35 µL of 1x TE (VWR International, Radnor, PA, USA) and were incubated at room temperature for 2 min. The plate was placed on the magnetic stand for 5 min, and 30 µL of clear supernatant was transferred to a new 96 well plate (V-bottom with lid, Costar). The plate was sealed with adhesive film (VWR International, Radnor, PA, USA) and stored at −20 °C until use.

PCR2: A second PCR with indexing primers was performed using a Nextera XT Index Kit v2 (Illumina, San Diego, CA, USA). Each 50 µL reaction contained 5 µL purified PCR1 product, 10 µL UltraClean PCR Water, 5 µL Nextera XT index primer 1, 5 µL Nextera Index primer 2, and 25 µL 2x Kappa HiFi HotStart Ready Mix. The following thermocycler conditions were used: initial denaturation at 95 °C for 3 min, followed by 8 cycles of denaturation at 95 °C for 30 s, annealing at 55 °C for 30 s, and extension at 72 °C for 30 s, with one final extension step at 72 °C for 5 min. The PCR reactions were purified with AMPure XP beads (Beckman Coulter, Pasadena, CA, USA) into a total volume of 20 µL of 1x TE. Samples were stored at −20 °C until use.

PCR2 clean up: PCR2 reactions were purified using AMPure XP beads (Beckman Coulter). Beads were vortexed, and 56 µL was added to each well of a 0.8 mL 96 well plate (Thermo Scientific). The remaining procedures were similar to PCR1 clean up as described above. The plate was placed on a magnetic stand for 5 min, and 20 µL of clear supernatant was transferred to a new 96 well plate (V-bottom with lid, Costar, Arlington, VA, USA).

***PCR product assessment*:** Purified amplicons were assessed for quality and quantity before sequencing on the Illumina MiSeq platform. DNA quantity was assessed using Picogreen assay as described above. DNA quality was assessed by running samples on a 2% gel with 2 µL of 6X gel loading dye, 6 µL of PCRII product, and 5 µL of 100 bp plus DNA ladder. A PCR product of 250 bp was observed for ITS, and a PCR product of 650 bp was observed for 16S. Sequencing was performed using the Illumina MiSeq platform (Illumina, San Diego, CA, USA) as described previously [19].

***Bioinformatics and Statistical Analysis*:** Paired-end sequences of the ITS1 region and the hypervariable regions of the 16S were joined using Paired-End reAd mergeR (PEAR v. 0.9.6) [22]. These sequences were filtered for sequence quality using Fastx-Toolkit (v. 0.013) to isolate reads with 90% of their bases with a score higher than Q30. Primer sequences were trimmed based on the length of the forward and reverse sequencing primers, and sequences were then clustered at 97% identity against the ISHAM database [23] for ITS1 sequences and against the HOMD (v. 15.1) for 16S sequences with BLAST (v. 4.0) [24] for species-level identification. Samples with hit counts lower than 3000 were removed in downstream analyses. We used relative abundance, within the sample, to characterize the community composition of microbiome and mycobiome. Wilcoxon rank-sum test [25] was used to evaluate the significance of differences among relative abundances of OTUs, adjusting for multiple testing correction using the Benjamini–Hochberg method [26].

## 3. Results

***Selection of lysing matrix:*** In this study, DNA yield in the pooled saliva samples ranged from 3.42 to 4.57 ng/µL for three matrices, and this was deemed adequate for amplicon sequencing. Qualitatively, lysing matrix A provided reproducible results with sequencing for fungal and bacterial ecology in replicates. *Candida albicans* and *Candida dublinensis* were predominantly noted in all replicates, and their relative abundance varied from 20 to 80% and 20 to 78%, respectively. Other fungi comprised less than 1% of the mycobiome. For bacteria, various species of genus *Veillonella* were found to be dominant in abundance and comprised between 36 and 45% of the sample microbiome. *Veillonella atypica* and *Veillonella dispar* were the most common species in the genus and comprised between 28 and 36% in replicates. Lysing matrix A was used for individual samples considering the quantitative and qualitative reproducibility seen in pooled saliva samples.

***Microbial and fungal ecology*:** Our study showed that across all samples (*n* = 12), a total of 80 bacterial genera and 375 bacterial species were identified. *Porphyromonas gingivalis* was noted in five samples and only in samples of participants with severe periodontitis. No *Porphyromonas gingivalis* was noted in samples of participants with mild periodontitis or periodontal health. *Tannerella forsythia* was noted in five samples of participants with mild and severe periodontitis but not in samples from participants with periodontal health. The average number of bacterial species per sample was reduced from periodontitis to health (137.50), with more bacterial species per sample in severe periodontitis (198.57) than in mild periodontitis (141.33).

In our study, across all samples (*n* = 12), a total of 39 fungal species was identified. Six *Candida* species were identified, including *albicans, dublinensis, intermedia, parapsilosis, tropicalis, and zeylanoides*. All samples contained *C. albicans* and *C. dublinensis*. *C. parapsilosis* and *C. zeylanoides* were not found in samples of participants with periodontal health. On average, eleven fungal species were identified per sample in participants with periodontal health, eight in those with mild periodontitis, and thirteen in participants with severe periodontitis.

**Figure 2:**
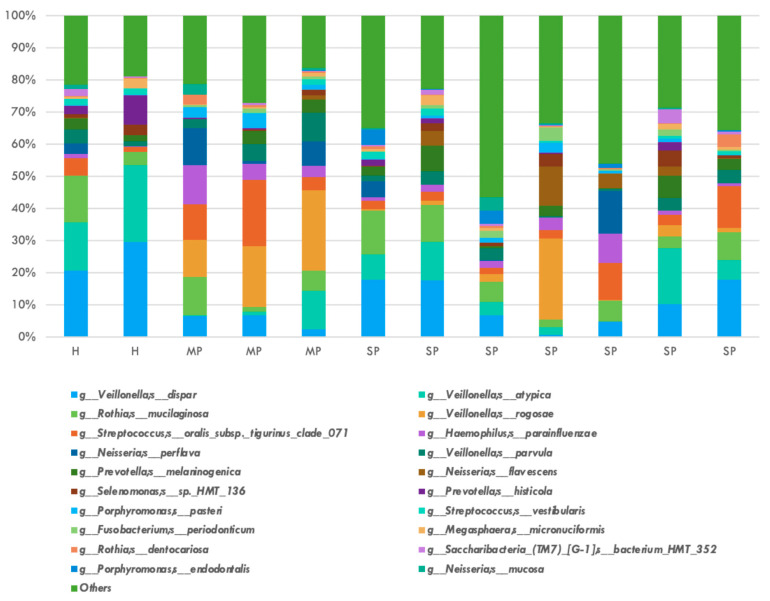
Top 20 bacterial species by relative abundance after 16S amplicon sequencing: *Veillonella dispar* and *Veillonella atypica* were most abundant, and the red complex bacteria were not noted in the top 20 bacterial species by relative abundance.

**Figure 3:**
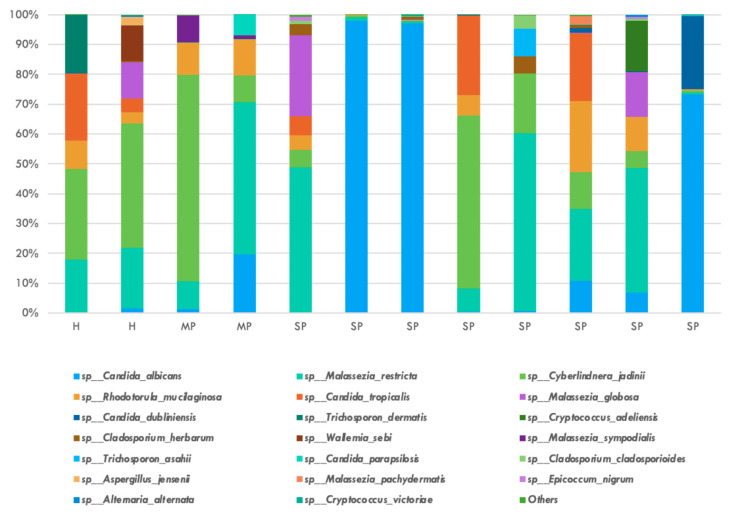
Top 20 fungal species by relative abundance after ITS amplicon sequencing: *Candida albicans* was the most abundant fungal species, and three other Candida species were noted in the top 20, namely *tropicalis, dublinensis*, and *parapsilosis*.

The relative abundance of the top twenty bacterial species is shown in Figure 2. *P. gingivalis, T. denticola*, and *T. forsythia* were not represented in the top twenty species by relative abundance among all participant groups in this study. *Veillonella* genus represented the top two most frequent bacterial species, with *dispar* and *atypica* having average relative abundance of 0.1175 and 0.0852, respectively. The relative abundance of the top twenty fungal species is shown in Figure 3. Four *Candida* species were identified in the top twenty fungal species: *albicans, tropicalis, dublinensis*, and *parapsilosis*. *Candida albicans* represented the most frequently found fungi with a relative abundance of 0.2581.

**Figure 4:**
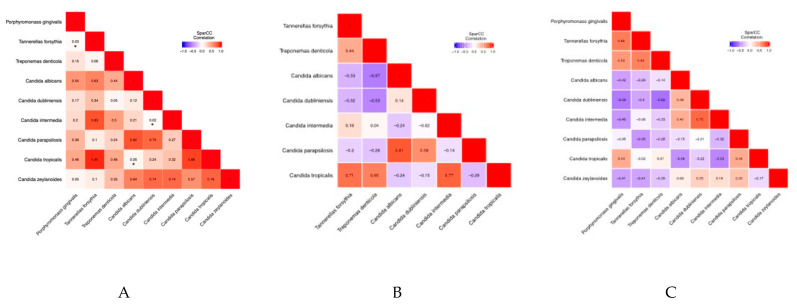
Correlation between red complex bacteria and Candida species in (**A**) all groups, (**B**) healthy and mild periodontitis, and (**C**) severe periodontitis. * Statistical significance with *p* < 0.05.

***Correlation between red complex bacteria and Candida:*** Table 2 shows pairwise correlation of red complex bacteria with *Candida* species for all groups in this study. Statistically significant correlations were noted between *P. gingivalis* and *T. forsythia* (correlation coefficient: 0.537, *p*-value: 0.026), *C. tropicalis* and *C. albicans* (correlation coefficient: −0.498, *p*-value: 0.049), and *C. intermedia* and *C. dublinensis* (correlation coefficient: 0.602, *p*-value: 0.019) (Table 3). There were no significant correlations between *Candida* species and red complex bacteria in healthy participants and those with mild periodontitis (Table 4 and Table 5). Further, *P. gingivalis* was not found in any samples from the periodontally healthy and mild periodontitis groups. Table 6 shows pairwise correlation of red complex bacteria with *Candida* species in the group with severe periodontitis. Statistically significant correlations were noted between *P. gingivalis* and *T. forsythia* (correlation coefficient: 0.663, *p*-value: 0.038), *T. forsythia* and *T. denticola* (correlation coefficient: 0.642, *p*-value: 0.041), and *C. intermedia* and *C. dubliniensis* (correlation coefficient: 0.754, *p*-value: 0.029) (Table 7). An overview of heatmaps (Figure 4) shows a trend towards negative correlations between red complex bacteria and *Candida* species.

## 4. Discussion

The oral microbiome is a complex ecosystem of diverse bacteria, fungi, and viruses, which has a fundamental influence on the health and disease states of humans [27,28]. While the bacterial microbiome has been well studied, resulting in well-annotated databases, the fungal microbiome or mycobiome is understudied due to several reasons. Fungi are only a small proportion (<0.1%) of the entire microbiome [29]. The isolation of genomic DNA from fungi is difficult due to tough cell walls, and many fungal species remain uncultivable [19,24,28]. Furthermore, fungal databases have not been annotated well, resulting in errors and redundancies in identification [30].

The importance of fungi has increased recently due to their association with inflammatory bowel disease, Crohn’s disease, and chronic respiratory diseases [31]. These diseases are characterized by fungi–bacteria interactions via the formation of a biofilm, which benefits fungi in producing virulence factors and bacteria in attaining antimicrobial resistance [27]. The hyphae produced by fungi become skeletal for the adherence of bacteria and development of inter-kingdom biofilms. A great paradigm for such fungi is *Candida albicans*, which is ubiquitously present in various niches in humans, including the oral cavity.

In our study, ITS sequencing identified various fungal genera (*n* = 18) and species (*n* = 39) across all groups—healthy, mild periodontal disease, and severe periodontal disease. The subjects of this study did not have any underlying systemic condition. Thus, a targeted approach for sequencing should be considered to gain a qualitative understanding of mycobiome as opposed to an exploratory approach. In contrast to our study, Zhu et al. obtained data from 3346 oral metagenomic samples, including saliva and tongue swabs, and did not find any fungi in their analysis, a rather surprising result [32]. They attribute this result to a lack of fungi in the samples. However, it is more likely that their genomic DNA extraction methods were unable to release fungal DNA from their samples, as fungi are present in extremely low quantities (<0.1%) as compared to bacteria and are also difficult to lyse. Furthermore, Zhu et al. also used metagenomic sequencing instead of ITS sequencing for their analysis. In our study which utilized ITS sequencing, Candida was the most abundant genus identified, with four species in the top 20 by relative abundance: *albicans*, *tropicalis*, *dublinensis*, and *parapsilosis.* While variability was observed for *Candida* species across samples, a significant correlation was observed between these species in this study. A synergistic relationship between *Candida* species has been demonstrated previously in an in vitro system [33]. Of the four most common pathogenic fungi, *Candida, Aspergillus*, *Fusarium*, and *Cryptococcus*, *Fusarium* was not detected in any sample. However, *Aspergillus* was detected in one healthy and severe periodontitis sample each, and *Cryptococcus* was detected in nine participants (one healthy, one mild periodontitis, and seven severe periodontitis). The presence of *Cryptococcus* in all samples in the severe periodontitis group was an unexpected finding as *Crytptococcus* in the oral cavity is expected in immunocompromised patients [34,35]. However, it has been isolated from root canals with apical periodontitis [36] in otherwise healthy patients and noted in 20% of healthy patients in a study by Ghannoum et al. [13]. It is likely that *Cryptococcus* is found in low numbers as a commensal in the oral cavity but can act opportunistically in local and systemic disease states. Further, various *Malassezia* species were noted across samples in all groups. *Malassezia* is associated with various dermatologic disorders [37] but was proposed for inclusion in the human basal oral mycobiome, as it was noted in all samples in a study conducted by Dupuy et al. [38]. Microbes can be flexible in their adaptation to various body sites, but a new environment can influence their physiologic state [39]. A comparative genomics approach to understand transcriptional differences between newly discovered oral fungal species otherwise native to other body sites can aid in a mechanistic understanding of host–microbe interactions. The 16s rRNA sequencing identified various bacterial genera (*n* = 8) and species (*n* = 375) across all groups. Red complex bacteria, *P. gingivalis, T. forsythia*, and *T. denticola,* were most abundant in the severe periodontitis group. However, they were not noted in the top 20 bacterial species by relative abundance. This is expected, as keystone species such as *P. gingivalis* have a disproportionate contribution in disease pathogenesis when compared to their low abundance [40]. There was significant correlation between red complex bacteria in the severe periodontitis group, but they were negatively correlated with Candida in the mild and severe periodontitis groups. Since red complex bacteria and Candida species have both been associated with periodontal disease [14,41], these results may seem counter intuitive. However, a recent systematic review on the relationship between Candida and periodontitis highlighted high heterogeneity in study design, sampling methods, and detection methods [42]. Whether oral fungal isolates are a primary step for opportunistic bacterial infection or increased abundance of periopathogenic bacteria could downregulate fungal species once a bacterial community is established remains to be established. It is possible that fungal species associated with periodontitis do not necessarily contribute to the disease state but are less likely to populate with a healthy oral microbiome. The pathogenic contribution of the bacterial–fungal interactions is unclear with regards to periodontitis.

Hajishengallis et al. proposed a polymicrobial synergy and dysbiosis (PSD) model in which the disease state would result from a combination of certain microbiota and specific genes [43]. It is likely that this polymicrobial contribution will include fungal–bacterial interactions towards a synergistic and dysbiotic state. The interactions of *C. albicans* with various oral bacteria have been well documented [27,44,45,46,47]. *C. albicans* interacts with *Streptococcus oralis*, resulting in increased biofilm formation, disruption of the epithelial junctions, and increased tissue invasion [48]. Additionally, *C. albicans* interaction with *Streptococcus mutans* results in increased biofilm formation, causing an increased incidence of early childhood caries [49]. Furthermore, *C. albicans* interactions with *Fusobacterium nucleatum* aids in the immune evasion of the latter [45]. Such interkingdom interactions warrant the study of the microbiome and mycobiome within the same sample, and data from such studies may help in improving our understanding of correlations between specific fungal and bacterial species and further aid in the development of targeted hypotheses.

## 5. Conclusions

This pilot study characterized the salivary mycobiome and microbiome of healthy and periodontitis participants through targeted sequencing and synchronous sample preparation. In our study, we showed that positive correlation was observed between members of the red complex bacteria, and a negative correlation was observed between red complex bacteria and Candida species, which is contrary to previous studies. Although the mycobiome is only less than 0.1% of the total oral microbiome, our study has been successful in not only identifying fungi in saliva samples but also has developed models for analysis of correlation between bacteria and fungi. This is highly applicable in monitoring the dysbiosis of the oral microbiome and will be useful in developing microbial diagnostic methods using saliva as a sample. However, a major limitation of this study is the small sample size. Furthermore, subgingival plaque samples are more representative of the periodontal microbiome. Thus, our future studies will focus on not only a larger sample size but also perform a comparative analysis between ITS sequencing and metagenomic sequencing of saliva samples and subgingival plaque samples to understand bacterial and fungal correlations with greater confidence in periodontal disease patients.

## Figures and Tables

**Table 1:** PCR conditions and primers for the mycobiome and microbiome sequencing.

Type of Sequencing	Mycobiome (Fungi)	Microbiome (Bacteria)
**Region of** **Amplification**	Internal transcriber sequence ITS1 region	16s rRNA V3-V4 regions
**Primer Sequences**	ITS1F-CTTGGTCATTTAGAGGAAGTAA ITS1R-GCTGCGTTCTTCATCGATGC	F-AGAGTTTGATCCTGGCTCAG R-ACGGCTACCTTGTTACGACTT
**PCR 1 Conditions**	95 °C for 3 min 30 s 95 °C 30 s 50 °C Repeat 25 cycles 30 s 72 °C 5 min 72 °C	95 °C for 3 min 30 s 95 °C 45 sec 55 °C Repeat 25 cycles 30 s 72 °C 5 min 72 °C
**PCR 2 Conditions**	3 min 95 °C 30 s 95 °C 30 s 55 °C Repeat 8 cycles 30 s 72 °C 5 min 72 °C	3 min 95 °C 30 s 95 °C 30 s 55 °C Repeat 8 cycles 30 s 72 °C 5 min 72 °C

**Table 2:** Pairwise correlation of red complex bacteria and Candida species for all groups.

Correlation Coefficients	*Porphyromonas gingivalis*	*Tannerella forsythia*	*Treponema denticola*	*Candida albicans*	*Candida dubliniensis*	*Candida intermedia*	*Candida parapsilosis*	*Candida tropicalis*	*Candida zeylanoides*
***Porphyromonas gingivalis***	1	0.53731278	0.333041183	−0.151767367	−0.351388456	−0.299375574	−0.20625247	0.183436106	−0.339953215
***Tannerella forsythia***	0.53731278	1	0.441430423	−0.125639606	−0.253119816	−0.047184177	−0.399397455	−0.015253018	−0.30703011
***Treponema denticola***	0.333041183	0.441430423	1	−0.202235196	−0.489641502	−0.149063176	−0.260111701	0.185394178	−0.158882632
***Candida albicans***	−0.151767367	−0.125639606	−0.202235196	1	0.425660058	0.298167467	0.067746325	−0.498225752	−0.102562846
***Candida dubliniensis***	−0.351388456	−0.253119816	−0.489641502	0.425660058	1	0.602334681	0.07850208	−0.31303916	0.072137738
***Candida intermedia***	−0.299375574	−0.047184177	−0.149063176	0.298167467	0.602334681	1	−0.238488494	−0.235386894	0.066417965
***Candida parapsilosis***	−0.20625247	−0.399397455	−0.260111701	0.067746325	0.07850208	−0.238488494	1	0.039675969	0.10113917
***Candida tropicalis***	0.183436106	−0.015253018	0.185394178	−0.498225752	−0.31303916	−0.235386894	0.039675969	1	0.074242306
***Candida zeylanoides***	−0.339953215	−0.30703011	−0.158882632	−0.102562846	0.072137738	0.066417965	0.10113917	0.074242306	1

**Table 3:** *p*-Values for correlation of red complex bacteria and Candida species for all groups.

*p*-Values	*Porphyromonas gingivalis*	*Tannerella forsythia*	*Treponema denticola*	*Candida albicans*	*Candida dubliniensis*	*Candida intermedia*	*Candida parapsilosis*	*Candida tropicalis*	*Candida zeylanoides*
***Porphyromonas gingivalis***	1	0.026	0.148	0.536	0.173	0.2	0.388	0.458	0.05
***Tannerella forsythia***	0.026	1	0.055	0.633	0.342	0.832	0.098	0.946	0.103
***Treponema denticola***	0.148	0.055	1	0.436	0.054	0.503	0.243	0.456	0.35
***Candida albicans***	0.536	0.633	0.436	1	0.119	0.206	0.821	0.049	0.636
***Candida dubliniensis***	0.173	0.342	0.054	0.119	1	0.019	0.748	0.244	0.737
***Candida intermedia***	0.2	0.832	0.503	0.206	0.019	1	0.266	0.323	0.735
***Candida parapsilosis***	0.388	0.098	0.243	0.821	0.748	0.266	1	0.875	0.573
***Candida tropicalis***	0.458	0.946	0.456	0.049	0.244	0.323	0.875	1	0.759
***Candida zeylanoides***	0.05	0.103	0.35	0.636	0.737	0.735	0.573	0.759	1

Shaded area represents *p* < 0.05.

**Table 4:** Pairwise correlation of red complex bacteria and Candida species for healthy and mild periodontitis groups.

Correlation Coefficients	*Tannerella forsythia*	*Treponema denticola*	*Candida albicans*	*Candida dubliniensis*	*Candida intermedia*	*Candida parapsilosis*	*Candida tropicalis*
***Tannerella forsythia***	1	0.43774298	−0.333843702	−0.316563422	0.1799742	−0.201116436	0.706151712
***Treponema denticola***	0.43774298	1	−0.570547057	−0.533947765	0.036642522	−0.255673848	0.651375479
***Candida albicans***	−0.333843702	−0.570547057	1	0.142774181	−0.241524058	0.811733249	−0.236286454
***Candida dubliniensis***	−0.316563422	−0.533947765	0.142774181	1	−0.020422605	0.578807206	−0.151123919
***Candida intermedia***	0.1799742	0.036642522	−0.241524058	−0.020422605	1	−0.143151822	0.769874961
***Candida parapsilosis***	−0.201116436	−0.255673848	0.811733249	0.578807206	−0.143151822	1	−0.285399967
***Candida tropicalis***	0.706151712	0.651375479	−0.236286454	−0.151123919	0.769874961	−0.285399967	1

**Table 5:** *p*-Values for correlation of red complex bacteria and Candida species for healthy and mild periodontitis groups.

*p*-Values	*Tannerella forsythia*	*Treponema denticola*	*Candida albicans*	*Candida dubliniensis*	*Candida intermedia*	*Candida parapsilosis*	*Candida tropicalis*
***Tannerella forsythia***	1	0.333	0.442	0.528	0.712	0.541	0.07
***Treponema denticola***	0.333	1	0.239	0.316	0.932	0.401	0.081
***Candida albicans***	0.442	0.239	1	0.781	0.566	0.075	0.537
***Candida dubliniensis***	0.528	0.316	0.781	1	0.964	0.132	0.736
***Candida intermedia***	0.712	0.932	0.566	0.964	1	0.666	0.067
***Candida parapsilosis***	0.541	0.401	0.075	0.132	0.666	1	0.296
***Candida tropicalis***	0.07	0.081	0.537	0.736	0.067	0.296	1

**Table 6:** Pairwise correlation of red complex bacteria and Candida species for severe periodontitis groups.

Correlation Coefficients	*Porphyromonas gingivalis*	*Tannerella forsythia*	*Treponema denticola*	*Candida albicans*	*Candida dubliniensis*	*Candida intermedia*	*Candida parapsilosis*	*Candida tropicalis*	*Candida zeylanoides*
***Porphyromonas gingivalis***	1	0.663026251	0.533469205	−0.416165635	−0.577078078	−0.455449694	−0.064539964	0.435096681	−0.411388874
***Tannerella forsythia***	0.663026251	1	0.642781964	−0.291249055	−0.399786984	−0.057060816	−0.464981704	−0.015279857	−0.411604834
***Treponema denticola***	0.533469205	0.642781964	1	−0.139752468	−0.615080092	−0.226454353	−0.277915645	0.065709903	−0.262961763
***Candida albicans***	−0.416165635	−0.291249055	−0.139752468	1	0.376787143	0.415429972	−0.149909207	−0.480294922	0.085956511
***Candida dubliniensis***	−0.577078078	−0.399786984	−0.615080092	0.376787143	1	0.753572716	−0.011325989	−0.223835122	0.251957054
***Candida intermedia***	−0.455449694	−0.057060816	−0.226454353	0.415429972	0.753572716	1	−0.32434038	−0.534608342	0.175364742
***Candida parapsilosis***	−0.064539964	−0.464981704	−0.277915645	−0.149909207	−0.011325989	−0.32434038	1	0.441652891	0.317988583
***Candida tropicalis***	0.435096681	−0.015279857	0.065709903	−0.480294922	−0.223835122	−0.534608342	0.441652891	1	−0.169806535
***Candida zeylanoides***	−0.411388874	−0.411604834	−0.262961763	0.085956511	0.251957054	0.175364742	0.317988583	−0.169806535	1

**Table 7:** *p*-Values for correlation of red complex bacteria and Candida species for severe periodontitis groups.

*p*-Values	*Porphyromonas gingivalis*	*Tannerellas forsythia*	*Treponemas denticola*	*Candida albicans*	*Candida dubliniensis*	*Candida intermedia*	*Candida parapsilosis*	*Candida tropicalis*	*Candida zeylanoides*
***Porphyromonas gingivalis***	1	0.038	0.112	0.246	0.101	0.184	0.837	0.186	0.132
***Tannerellas forsythia***	0.038	1	0.041	0.44	0.327	0.866	0.15	0.963	0.141
***Treponemas denticola***	0.112	0.041	1	0.739	0.073	0.523	0.376	0.863	0.356
***Candida albicans***	0.246	0.44	0.739	1	0.369	0.253	0.698	0.153	0.801
***Candida dubliniensis***	0.101	0.327	0.073	0.369	1	0.029	0.977	0.626	0.479
***Candida intermedia***	0.184	0.866	0.523	0.253	0.029	1	0.295	0.084	0.518
***Candida parapsilosis***	0.837	0.15	0.376	0.698	0.977	0.295	1	0.164	0.303
***Candida tropicalis***	0.186	0.963	0.863	0.153	0.626	0.084	0.164	1	0.616
***Candida zeylanoides***	0.132	0.141	0.356	0.801	0.479	0.518	0.303	0.616	1

## Data Availability

The raw data supporting the conclusions of this article will be made available by the authors on request.
